# Nurse-like cells mediate ibrutinib resistance in chronic lymphocytic leukemia patients

**DOI:** 10.1038/bcj.2015.74

**Published:** 2015-10-02

**Authors:** F Boissard, J-J Fournié, A Quillet-Mary, L Ysebaert, M Poupot

**Affiliations:** 1 Centre de Recherches en Cancérologie de Toulouse, INSERM UMR1037, Université Toulouse III Paul-Sabatier, ERL 5294 CNRS, Toulouse, France; 2 Laboratoire d'Excellence 'TOUCAN', Programme Hospitalo-Universitaire en Cancérologie CAPTOR, Institut Carnot Lymphome CALYM, Pierre-Benite, France; 3 Institut Universitaire du Cancer de Toulouse—Oncopole, CHU de Toulouse, France

Chronic lymphocytic leukemia (CLL) is one of the most common B-cell malignancies in adults, characterized by an accumulation of monoclonal CD5^+^ mature B-cells in lymphoid tissues and peripheral blood (PB). Clonal expansion and invasive migration typically lead to CLL cell involvement in the lymph nodes (LNs), spleen and bone marrow (BM).^1^

Signaling from the B-cell antigen receptor regulates multiple cellular processes in CLL cells, such as proliferation, differentiation, apoptosis and cell migration.^2^ Bruton tyrosine kinase (BTK), a member of the Tec kinase family, is a signaling molecule positioned early on the B-cell antigen receptor signaling cascade, in close proximity to Syk and phosphoinositide 3-kinase delta (PI3Kδ). Ibrutinib is a selective and irreversible BTK inhibitor which inactivates this kinase through covalent binding to Cys-481 in its ATP-binding domain.^3^ It is widely used in CLL chemotherapy regimens and produces remarkable initial responses in patients with relapsed and refractory CLL. Indeed, ibrutinib not only induces early CLL cell apoptosis in tissues, but also the redistribution of tissue-resident CLL cells into the PB with a rapid resolution of enlarged LNs, the improvement of cytopenia (potentially due to egress from the BM) and a surge in lymphocytosis.^4^ The emergence of ibrutinib-resistant BTK mutants has been observed in a subset of patients with prolonged exposure to this drug, although such mutants alone cannot account for the rapid disease progression and lack of complete response seen in virtually all CLL patients.^5^ The tumor microenvironment (TME) has been widely shown to be critical for tumor cell survival, chemoresistance, homing and proliferation.^6, 7^ The TME is a complex milieu made up of extracellular matrix, chemokines, cytokines, non-malignant cells including CD4^+^ helper T-cells, mesenchymal stromal cells and nurse-like cells (NLC).^8, 9, 10^ The persistence of CLL TME interactions despite ongoing therapy may contribute to relapse and thus could have a role in ibrutinib resistance. Although the induction of CLL apoptosis has been reported with high concentrations of ibrutinib despite coculture with NLC, at clinically achievable concentrations, that is, 0.1–0.4 μm, ibrutinib-induced apoptosis in CLL cells was blocked *in vitro* by HS-5 or NKTert stromal cell lines.^11, 12^ The CD68^+^ CD163^+^ NLC subset of macrophage cells have been described as tumor-associated macrophages in CLL and are found in various tumoral niches (LNs, spleen and BM).^10, 13^ Little is known on how anti-leukemic agents impact on drug-exposed patient monocytes *in vivo*, and whether this translates into a perturbed NLC differentiation program and reduced CLL survival *in vitro*. In this work, we raise the hypothesis that ibrutinib resistance arises from its lack of efficiency in blocking CLL-monocyte interactions in ibrutinib-treated patients, leaving NLC induction and pro-survival capacities unaffected and thus emphasizing the role of the TME in persistent residual disease.

In this study, we report for the first time that (i) ibrutinib does not induce detectable NLC egress into the bloodstream, (ii) monocytes remain miseducated to differentiate into fully functional NLC by CLL cells in ibrutinib-treated patients and (iii) these NLC efficiently promote CLL survival.

Ibrutinib induces an egress of CLL cells from their resident tissue niches into the blood^4^ therefore we first wondered if such a relocalization would also be observed for NLC. We analyzed freshly isolated PB mononuclear cells (PBMC) from ibrutinib-exposed relapsed CLL patients (420 mg/d). As shown in Figure 1a, CD14^+^ monocytes did not express the NLC marker CD163 at the time of blood sampling, similar to what has been shown in untreated patients.^10^ This suggested that ibrutinib does not induce the egress of fully differentiated NLC from niches, leaving a potential capacity for their interaction with residual CLL cells. We then cultured PBMCs from ibrutinib-treated patients to assess NLC morphology and phenotype at day 15. Indeed, *in vitro* culture of PBMC from CLL patients lead to the differentiation of monocytes in NLC. NLC morphology did not differ between ibrutinib-exposed and non-exposed PBMCs (not shown). Accordingly, the expression levels of many cell surface markers were comparable, including CD14, CD68, CD163, CD33 and CD11b (Figure 1b).^10, 13^ To investigate further, we determined the functional capacity of NLC *in vitro* generated from PBMC of ibrutinib-treated patients. Interestingly, NLC obtained *in vitro* from ibrutinib-treated patients promoted *in vitro* CLL cell survival (Figure 1c) as well as that of NLC from untreated patients (Figure 1d). Moreover, in the absence of NLC, CLL cells cultivated from ibrutinib-treated patients were more sensitive to apoptosis compared with CLL cells from untreated patients (Figure 1d). Altogether, these results show that ibrutinib treatment does not alter the capacity of CLL cells to induce monocyte differentiation into NLC, suggesting that this process is BTK-independent.

Next, we evaluated the chemoprotective effects of NLC from untreated patients against a variety of anti-leukemic agents: rituximab (10 μg/ml), bendamustin (10 μm), dasatinib (30 nm), idelalisib (4 μm), venetoclax (ABT-199; 0.5 nm) and ibrutinib (0.5 μm). After a week of coculturing patient CLL cell with NLC, CLL viability was measured. To make sure that the *in vitro* data were relevant, drug concentrations were chosen according to the maximum achievable concentrations reported in clinical trials. At these selected doses, apoptosis was detectable for all anti-leukemic agents when CLL cells were treated without NLC, with the expected inter-patient heterogeneity (Figure 2a). However, apoptosis induced by ibrutinib was significantly rescued by coculture of CLL cells with NLC, a protective effect that was not observed with rituximab, bendamustin, dasatinib, venetoclax or idelalisib (Figure 2a). Interestingly, this protective effect of NLC seemed to be dependent on the concentration of ibrutinib used. Indeed, in accordance with the results of Herman *et al.,*^11^ NLC were unable to protect CLL cells against a high concentration of ibrutinib (10 μm), whereas at a clinically relevant concentration (0.5 μm) NLC induced ibrutinib resistance, as observed with a stromal cell line (Figure 2b).^12^ Finally, we tested the combination efficacy of bendamustin (10 μm) with idelalisib (4 μm) or ibrutinib (0.5 μm) on CLL cell death when cultured with or without NLC. The addition of idelalisib or ibrutinib improved cell death induced by bendamustin irrespective of NLC support (Figures 2c and d). Thus, the chemoprotective effect induced by NLC in ibrutinib-treated patients was lost upon cotreatment with bendamustin (Figure 2d).

Together, our *in vitro* experiments suggest that the pro-apoptotic effects of ibrutinib may be at least partially rescued by NLC present in the TME. Combination studies warrant further thorough analysis from *in vitro* coculture studies as our data support recent clinical evidence of the superiority of R-bendamustine-ibrutinib combination treatment in relapsed patients. Furthermore, our results show that venetoclax, a Bcl-2 antagonist with cytotoxic effects against CLL cells, is active in the presence of NLC, suggesting that this compound can effectively replace bendamustin in combination studies.

## Figures and Tables

**Figure 1:**
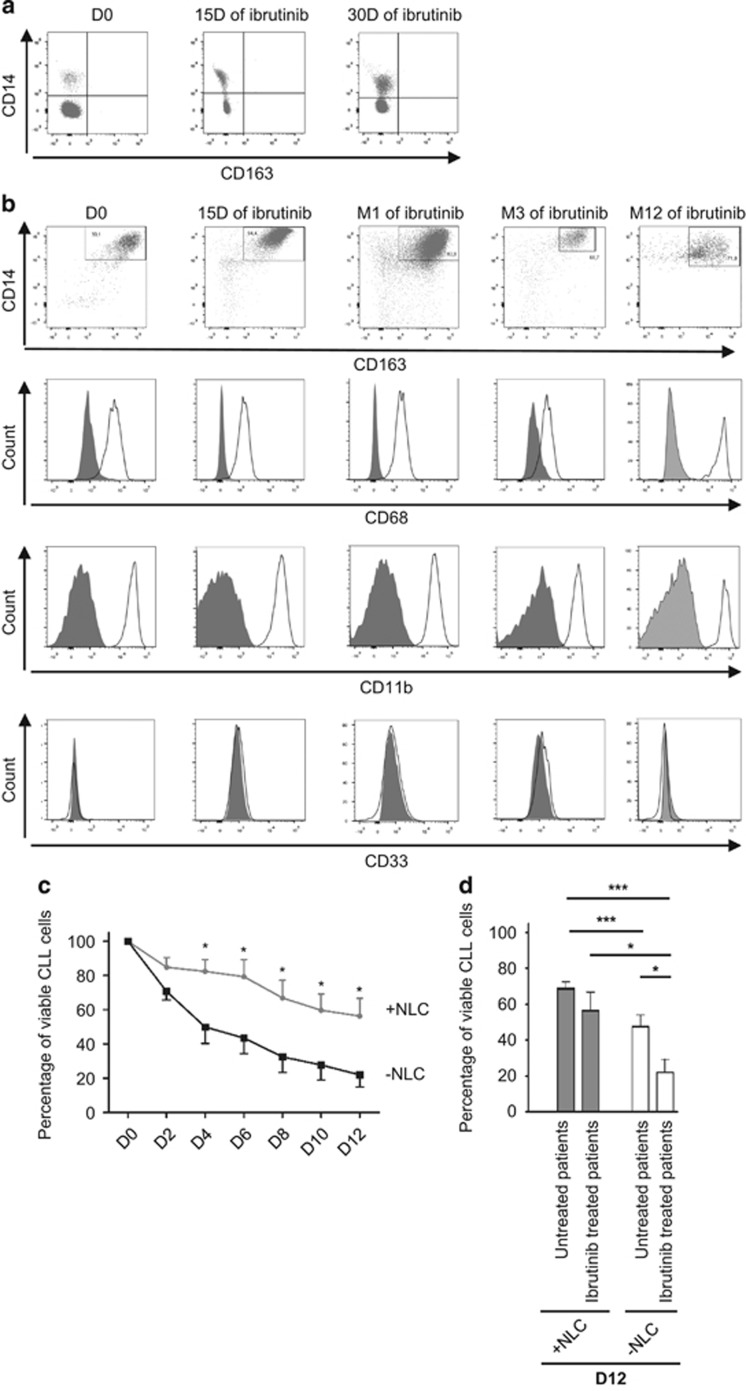
*In vivo* ibrutinib treatment does not hamper NLC phenotype and function. (**a**) NLC were absent from the PB during ibrutinib treatment at the time of blood sampling (left: before ibrutinib treatment, center and right: 15 days and 30 days of ibrutinib treatment, respectively). (Representative results from five independent experiments). (**b**) Flow cytometry analysis of the expression of CD14, CD163, CD68, CD11b and CD33 by NLC before (day 0, D0) or after ibrutinib treatment of the patient for the times shown (15 days or 1, 3 or 12 months), compared with isotype control (gray) after 15 days of differentiation. (Representative results from five independent experiments). (**c**) Percentage viability of CLL cells from ibrutinib-treated patients when cultured alone (black line) or with their own NLC (gray line). (Data taken from five independent experiments). (**d**) Percentage viability of CLL cells from untreated or ibrutinib-treated patients after 12 days of culture either alone or in the presence of NLC from untreated or ibrutinib-treated patients. (Data taken from five independent experiments for ibrutinib-treated patients, seven independent experiments for untreated patients). (*P<0.05 and ***P<0.001).

**Figure 2:**
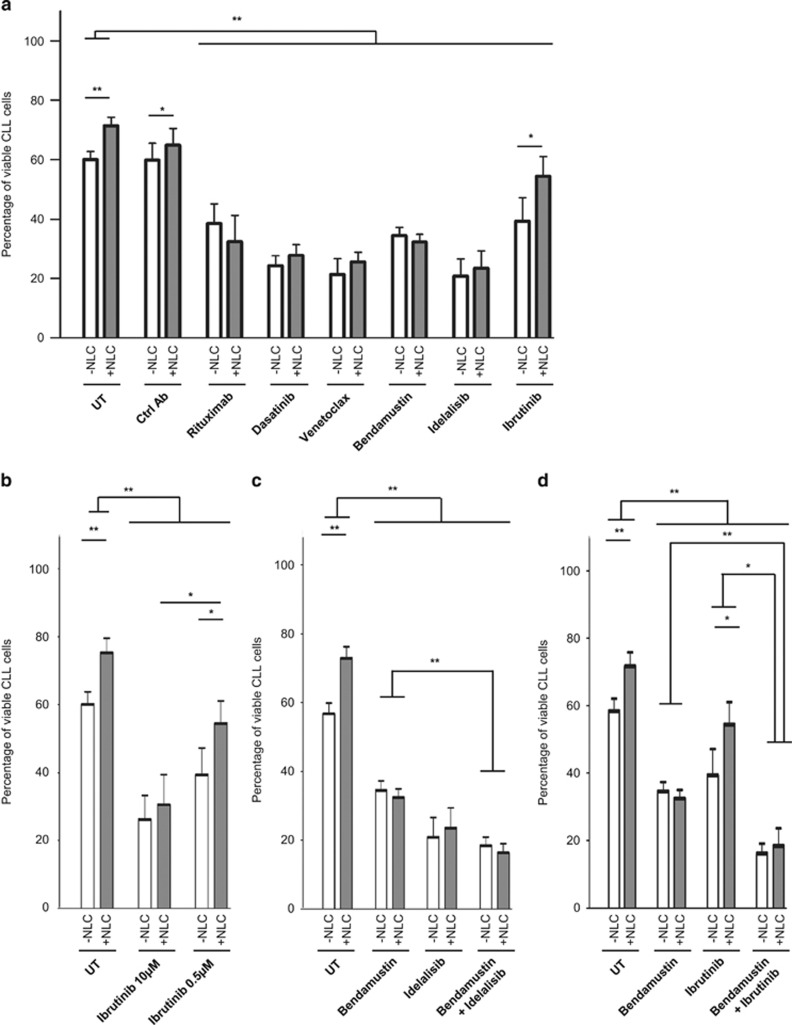
NLC protect CLL cell survival against ibrutinib at clinical concentrations *in vitro*. (**a**) Percentage viability of CLL cells after 7 days of culture either alone or in the presence of NLC treated or not (untreated, UT) with the indicated drugs. (**b**) Percentage viability of CLL cells after 7 days of culture either alone or in the presence of NLC treated or not (UT) with ibrutinib at the indicated concentrations. (Data taken from eight independent experiments for each indicated drug). (**c**) Percentage viability of CLL cells after 7 days of culture either alone or in the presence of NLC treated or not (UT) with bendamustin, idelalisib or both. (Data taken from eight independent experiments for each indicated drug). (**d**) Percentage viability of CLL cells after 7 days of culture either alone or in the presence of NLC treated or not (UT) with bendamustin, ibrutinib or both. (Data taken from eight independent experiments for each indicated drug). (*P<0.05 and **P<0.01).
